# Isolation of alkaliphilic calcifying bacteria and their feasibility for enhanced CaCO_3_ precipitation in bio‐based cementitious composites

**DOI:** 10.1111/1751-7915.13752

**Published:** 2021-02-25

**Authors:** Nafeesa Shaheen, Amna Jalil, Fazal Adnan, Rao Arsalan Khushnood

**Affiliations:** ^1^ NUST Institute of Civil Engineering (NICE) School of Civil and Environmental Engineering (SCEE) National University of Sciences and Technology (NUST) Sector H‐12 Islamabad 44000 Pakistan; ^2^ Atta‐ur‐Rahman School of Applied Biosciences (ASAB) National University of Sciences and Technology (NUST) Sector H‐12 Islamabad 44000 Pakistan

## Abstract

Microbially induced calcite precipitation (MICP), secreted through biological metabolic activity, secured an imperative position in remedial measures within the construction industry subsequent to ecological, environmental and economical returns. However, this contemporary recurrent healing system is susceptible to microbial depletion in the highly alkaline cementitious environment. Therefore, researchers are probing for alkali resistant calcifying microbes. In the present study, alkaliphilic microbes were isolated from different soil sources and screened for probable CaCO_3_ precipitation. Non‐ureolytic pathway (oxidation of organic carbon) was adopted for calcite precipitation to eliminate the production of toxic ammonia. For this purpose, calcium lactate Ca(C_3_H_5_O_3_)_2_ and calcium acetate Ca(CH_3_COO)_2_ were used as CaCO_3_ precipitation precursors. The quantification protocol for precipitated CaCO_3_ was established to select potent microbial species for implementation in the alkaline cementitious systems as more than 50% of isolates were able to precipitate CaCO_3_. Results suggested 80% of potent calcifying strains isolated in this study, portrayed higher calcite precipitation at pH 10 when compared to pH 7. Ten superlative morphologically distinct isolates capable of CaCO_3_ production were identified by *16SrRNA* sequencing. Sequenced microbes were identified as species of *Bacillus*, *Arthrobacter*, *Planococcus*, *Chryseomicrobium* and *Corynebacterium*. Further, microstructure of precipitated CaCO_3_ was inspected through scanning electron microscopy (SEM), X‐ray diffraction (XRD) and thermal gravimetric (TG) analysis. Then, the selected microbes were investigated in the cementitious mortar to rule out any detrimental effects on mechanical properties. These strains showed maximum of 36% increase in compressive strength and 96% increase in flexural strength. *Bacillus*, *Arthrobacter, Corynebacterium* and *Planococcus genera* have been reported as CaCO_3_ producers but isolated strains have not yet been investigated in conjunction with cementitious mortar. Moreover, species of *Chryseomicrobium* and *Glutamicibacter* were reported first time as calcifying strains.

## Graphical abstract

In the present study, alkaliphilic microbes were isolated from different soil sources and screened for probable CaCO_3_ precipitation. Non‐ureolytic pathway (oxidation of organic carbon) was adopted for calcite precipitation to eliminate the production of toxic ammonia. By performing SEM,XRD and TG analysis, precipitated powder was characterized as calcite. These isolated strains showed a maximum of 36% increase in compressive strength and 96% increase in flexural strength.

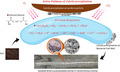

## Introduction

Profoundly, microbes transpire as a prospective aspirant of biomedicine, food preservation, waste management, agricultural and bio‐mineralization industries owing to their natural metabolic aptitude (Oren, 2010; Sarethy *et al*., 2011; Gurung et al., 2013). Particularly, bio‐mineralization is a primitive and evolving technique in construction and environmental industries as bioremediation, bio‐consolidation, bio‐rehabilitation and bio‐degradation (Tazaki, 2006; Li *et al*., 2010; Skuce *et al*., 2017). Bio‐mineralization instigates the crystallization of inorganic minerals of calcium that are proposed for rehabilitation of ornamental stones, consolidation of sand and crack remediation in the cementitious compounds (Ashurst and Dimes, 1990; Tiano *et al*., 1999; Al‐Thawadi, 2011; Kim *et al*., 2016). Remediation of cementitious compounds requires recurring maintenance as cracks are unavoidable in these structures (Kantzas *et al*., 1992, Jonkers, 2008, Adjoudj *et al*., 2014; Shaheen and Khushnood, 2018). At present, coherent self‐healing concrete system via intrusion of calcifying microbes is designed to impart self‐remediated mechanisms to avoid external repairs (Stocks‐Fischer *et al*., 1999; Ramakrishnan *et al*., 2001; Chaurasia *et al*., 2014). Moreover, the intrusion of microbes enhances the mechanical strength of the concrete matrix against abrasion hence improving the structural health of the system (Khushnood et al., 2018). However, the repairing efficiency by MICP depends upon bacterial strain, precipitation pathway, pH, temperature, relative humidity, dissolved inorganic carbon and nutrients availability (Shaheen *et al*., 2019). Heterotrophic alkaline soil bacteria are mostly responsible for MICP including *Bacillus* sp., *Pseudomonas* sp.*, Sporosarcina* sp.*, Pantoea* sp. and few others (Daskalakis *et al*., 2013; Cho *et al*., 2015). Besides inducing calcite precipitation, these bacteria provide sites of nucleation or calcium enrichment (De Muynck *et al*., 2010). Heterotrophic bacteria precipitate calcite using passive and active pathways (Castanier *et al*., 1999).

The passive pathway involves the nitrogen cycle (dissimilatory reduction of nitrate, ammonification of amino acids and degradation of urea or uric acid) and sulfur cycle (dissimilatory reduction of sulfate) (Castanier *et al*., 2000), while the active pathway involves oxidation of organic matter in the presence of gaseous or dissolved oxygen and calcium. However, the active pathway of calcite precipitation is rarely investigated as compared to passive systems (De Belie and Wang, 2016; Lors *et al*., 2017). The downside of the nitrogen cycle is its by‐product ammonia which is detrimental for the environment and concrete (Belie 2016, 2016). Both urease and urea cannot stay up to years in concrete eventually healing potential will be declined (Jonkers, 2008). The metabolic conversion of calcite by ureolytic bacteria is optimum at pH 9. However, the highly alkaline pH of cementitious matrix severely affects the precipitation potential (Jonkers and Schlangen, 2008; Dhami *et al*., 2013). Similarly, sulfate reductions involve the formation of hydrogen sulfide which can be converted into elemental sulfur or sulfuric acid (Castro Alonso *et al*., 2019). Both compounds have the potential to corrode the steel in addition to being detrimental to concrete health (Jonkers and Schlangen, 2008; Dhami *et al*., 2013). However, in the active pathway, oxidation of organic acids contributes to pH increase and the concentration level of dissolved inorganic carbon eventually turns out in more calcite precipitation (Braissant *et al*., 2002). In this approach, aerobic oxidation of organic acids releases CO_2_, hence promoting the production of CO_3_^2‐^ in the highly alkaline environment that converts into CaCO_3_ using a calcium source (Luo *et al*., 2018). The active pathway is more sustainable owing to the non‐appearance of ammonium and sulfide. However, it requires high concentrations of calcium sources, that may be costly and may cause the accumulation of a high level of salts in the concrete matrix Menon et al. (2019a,b). Emerging evidence suggested that high alkaline pH of cementitious systems barred the microbial endurance in the long term (Shaheen *et al*., 2018). Immobilization of bacteria has been suggested for enhancing the survival of bacterial species inside the cementitious systems (Van Tittelboom *et al*., 2010; Khaliq and Ehsan, 2016; Khushnood *et al*., 2019). However, immobilizing material imparts extra cost and sometimes may react and can kill the microbes in addition to their interaction failure with cementitious systems (Shaheen *et al*., 2019). However, for intrinsic self‐healing mechanisms both bacteria and filler precursor compounds need to be integrated into the material matrix (Jonkers and Schlangen, 2008). Dissolved organic carbon influences calcite precipitation as well. Bacteria have been used for CO_2_ sequestration. Self‐heling concrete influenced by microbes is an alternative method for the removal of CO_2_ from the environment. Microbes via the active pathway of conversion organic acid into calcite use dissolved organic carbon that was near the surface of the concrete. This method is safer and more eco‐friendly than conventional methods of sequestering CO_2_ from the atmosphere (Anbu *et al*., 2016). Researchers are probing for a solution to formulate efficient concrete healing systems having highly alkaliphilic bacteria to eradicate the need for immobilization and sustainable precipitation pathways.

The state of the art highlights the research inadequacy about bacterial survival along calcite deposition pathways. Frequently urea hydrolysis pathway has been adopted that is detrimental for the environment and concrete (De Belie and Wang, 2016). In this current research, the active metabolic pathway was adopted for bacterial isolation for the elimination of nitrogen and sulfur cycle species (Mors and Jonkers, 2012). Moreover, all bacteria were isolated at highly alkaline pH and healing precipitate was characterized by scanning electron microscopy (SEM), X‐ray diffraction (XRD), thermal gravimetry (TG) and energy dispersive X‐ray spectrometry (EDS) mapping.

## Results and discussions

### Characterization of soil samples

The physical and chemical properties of collected soil samples were determined (Supporting information). It was made sure that all soil samples were slightly alkaline because isolation of alkaliphilic bacteria was intended for the study.

### Screening for calcifying bacteria

**Fig. 1:**
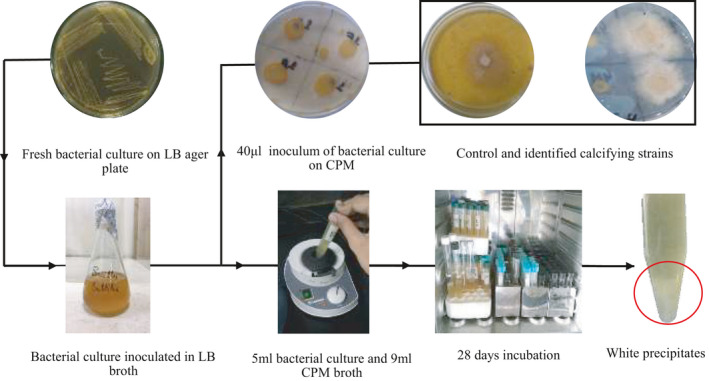
Process of identification of calcifying bacteria. (i) White halos represent the secreted calcite around the inoculation of calcifying bacteria in CPM plate. (ii) Red circle pointing the precipitated calcite at the bottom of falcon tube.

Almost 24 bacterial strains were able to grow and secrete calcite on CPM plate as well as in stationary falcon method. Calcifying bacterial strains produced halos around the bacterial inoculation on CPM agar plate along with crystal formation as shown in Fig. 1. These crystals were scraped off with the help of a spatula and dried. Chemical verification of dried powder was done by the addition of the acetic acid (CH_3_COOH) in the powder, causing effervescence thus proving the CaCO_3_ presence. Dried powder was dissolved in hydrochloric acid (HCl) for further confirming the presence of calcite (European Commission, 2012). Precipitated crystals at the bottom of the falcon (Fig. 1) were filtered, dried and chemical detection proved them to be calcite precipitates. These tests gave insight on calcifying strains but failed to rule out relative calcite precipitation.

### Quantification of calcite precipitation under different calcium sources

**Fig. 2:**
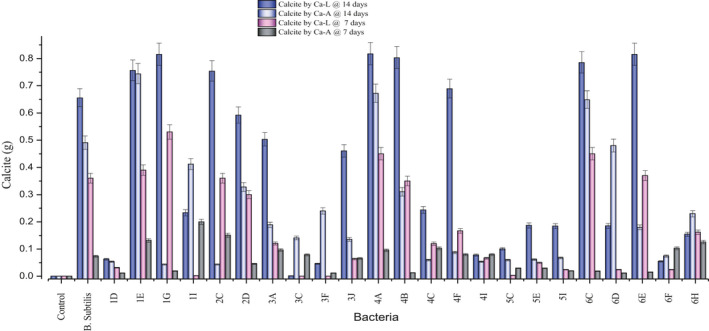
Quantification of calcite precipitation by calcifying bacterial strains under different calcium sources, i.e. calcium lactate and calcium acetate at the incubation age of 7 and 14 days.

Two types of calcium sources (calcium acetate and calcium lactate) were used for the calcification evaluation of bacterial strains at pH 10. The calcification potential of bacterial strains against calcium sources is represented in Fig. 2. Higher calcite precipitation was observed at the interval of 14 days of incubation as compared to 7 days of incubation. Calcite precipitation rate via active pathway is slower than urea hydrolysis (Reeksting et al., 2020). Almost all bacterial strains showed more calcite precipitation with calcium lactate as compared to calcium acetate, and a similar trend was observed by Jonker (2011). Additional calcite formation can be linked to release CO_2_ decomposition by bacterial metabolism as 1 mole of calcium lactate results in the formation of 1 mole of CaCO_3_ and 5 moles of CO_2_ according to Eq. 1. Dissolved CO_2_ reacts with water to form bicarbonate that later on converts into carbonate as explained in Eqs 2 to 4 (Xu *et al*., 2015; Wiktor and Jonkers, 2016). Moreover, calcium lactate has 13% elemental calcium ions, while calcium acetate has 25% calcium ions (Taylor et al., 1990; McEvoy, 2007). Some strains, i.e. 1E, 4A and 6C, depicted similar potential of calcite precipitation under both calcium sources. However, the formation of calcite via organic salt is a complex phenomenon that is not very well comprehended yet. Overall, 1G, 4A and 6E possessed higher calcite secretion potential. On the basis of calcite precipitation, 10 bacterial strains (1E, 1G, 2C, 2D, 3A, 4A, 4B, 4F, 6C and 6E) were selected for further analysis. Significance of experimental data was determined via one‐way ANOVA using *P* < 0.05 at confidence interval of 95%. Data were significant having *P* < 0.0001 along pooled standard deviation = 0.2142. *P* < 0.0001 confirmed the effect of calcium source on the calcite precipitation potential of bacteria (De Belie and Wang, 2016).

(1) $${\text{CaC}}_{6}H_{10}O_{6}{\;\text{+ 6O}}_{2}\to {\text{CaCO}}_{3}↓{\;\text{+ 5CO}}_{2}↑{\;\text{+ 5H}}_{2}O$$

(2) $${\text{CaC}}_{4}H_{6}O_{4}{\;\text{+ 4O}}_{2}\to {\text{CaCO}}_{3}↓{\;\text{+ 3CO}}_{2}↑{\;\text{+ 3H}}_{2}O$$

(3) $$H_{2}O\to H^{+}{\;\text{+ 2OH}}^{‐}$$

(4) $${\text{CO}}_{2}↑+H_{2}O\to H^{+}{\;\text{+ HCO}}_{3}^{‐}\to 2H^{+}+{\text{CO}}_{3}^{2‐}\to 2{\text{OH}}^{‐}+{\text{Ca}}^{2+}\to {\text{CaCO}}_{3}$$

### Effect of varying pH on calcite precipitation

**Fig. 3:**
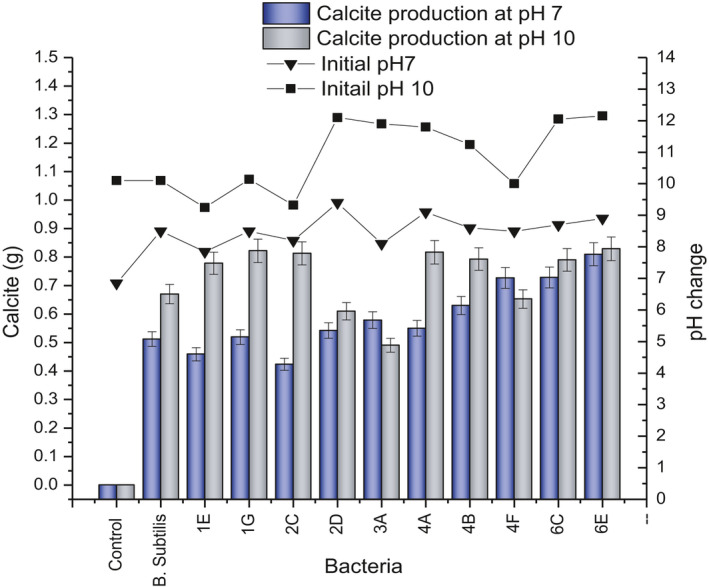
Calcite precipitation of selected calcifying strains at pH 7 and pH 10 with calcium lactate as calcium source.

The selected strains were tested for calcite production at pH 7 and pH 10 with calcium lactate as calcium source. Because cementitious system’s internal pH is higher, pH drops to 7 at the outer face where surface cracks occur (Lors *et al*., 2017). As calcite deposition by active pathway increased the system pH (Pan *et al*., 2019). So, change in pH at the end of incubation period was also monitored. The results of calcite precipitation of all strains at variable pH along pH change are illustrated in Fig. 3. Every bacterial strain increased the system pH differently and a similar trend was endorsed in literature (Silva‐Castro et al., 2015; Lors, *et al*., 2017). A maximum of pH changed 7 to 9.24 and 10 to 12.35 was observed in case of 2D strain. 1E strain slightly altered the system pH. Almost all calcifying strains portrayed higher calcite precipitation at pH 10 because higher pH accelerates the calcite precipitated rate in bacteria (Belie, 2016, 2016).

Strains 6C and 6E presented similar calcite precipitation at both values of pHs, while 3A and 4F showed higher activity at pH 7. 1E, 1G, 2C and 4A exhibited a noticeable reduction in calcification at pH 7. 2D and 4B depicted relative lower difference in calcite precipitation values at both pH values.

Overall, a 24.32% increment in calcite precipitation was observed in case of 1G strain at pH 10. 2C, 4A, 4B and 6E strains gave 22.3%, 23.1%, 18.35% and 23.4% more precipitation at pH 10 than reference strain. At pH 7, 6E strain scored 58.2% more precipitation than control followed by 6°C (42.1%), 4F (41.5%) and 4B (23.3%) respectively.

Significance of data was evaluated using paired *t*‐test, statistically. Data set was significant having *P*‐value of 0.0152 at confidence interval of 95%. T‐stat was also greater than T‐critical at α = 0.05 having pooled standard deviation = 0.2218. *P* < 0.0152 implied the effect of pH change on the calcite precipitation rate.

### Characterization of calcifying bacteria

Gram staining results showed that all the 10 strains were gram‐positive. Catalase, oxidase and urease test results are mentioned in Table S2. Eight strains are catalase‐positive and only 3A and 4F are catalase‐negative, whereas three strains 1G, 4A and 4B are oxidase‐positive and rest are oxidase‐negative. Only one strains 1E showed urease activity after 48 h. Most of the urease negative strains are being able to produce calcite via active pathway. Spore‐forming bacteria depict green colour, while non‐spore‐forming bacteria show pink colour under the optical microscope. Three strains 1G, 4A and 4B are characterized as spore formers while, rest as non‐spore formers. Spore‐forming bacteria are robust to survive in harsh environments (Jonkers, 2011).

### Phylogenetic analysis of the selected calcifying strains

**Fig. 4:**
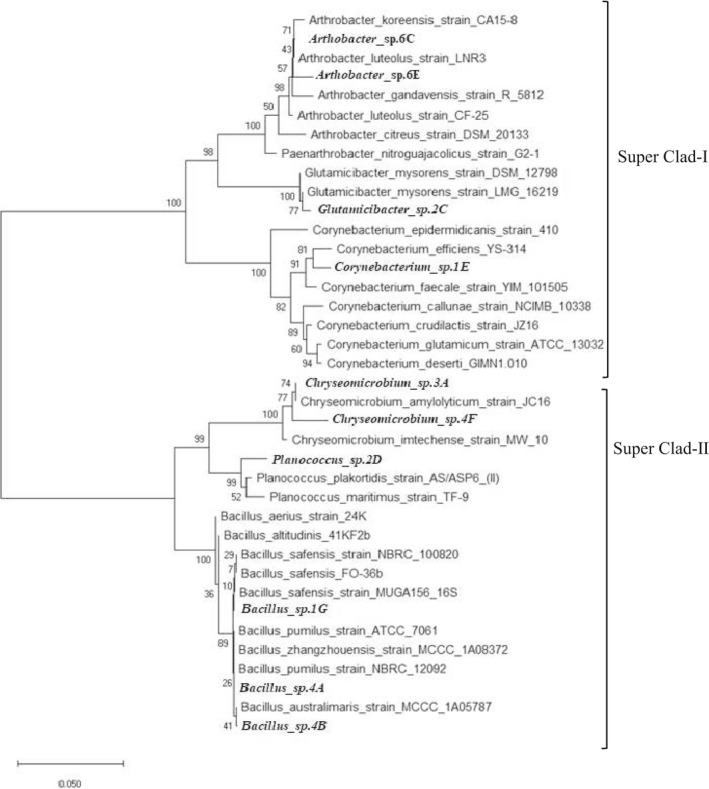
The evolutionary history was inferred using the neighbour‐joining method (Saitou and Nei, 1987). The optimal tree with the sum of branch length = 0.59515366 is shown. The evolutionary distances were computed using the Tamura–Nei method (Tamura and Nei, 1993) and are in the units of the number of base substitutions per site. The rate variation among sites was modelled with a gamma distribution (shape parameter = 1). This analysis involved 38 nucleotide sequences. All ambiguous positions were removed for each sequence pair (pairwise deletion option). There were a total of 1501 positions in the final data set. Evolutionary analyses were conducted in MEGA X (Kumar *et al*., 2018).

PCR product of *16S rRNA* gene of the ten higher calcite producing strains was sequenced to be sorted for sequence homology via BLAST. *Bacillus* sp. were most common in the identified strains, and the following prevailed species were *Chryseomicrobium* and *Arthrobacter*. *Bacillus* sp. and *Arthrobacter* sp. are the most abundant bacteria of soil (Cacchio *et al*., 2003). Rest belongs to the species of *Planococcus, Glutamicibacter* and *Corynebacterium*. Strain 1E was 98.18% identical to the *Corynebacterium efficiens* strain YS‐314 sequence. Strains 1G, 4A and 4B were 98.92%, 99.44% and 99.63% identical to *Bacillus safensis* MUGA 156, *Bacillus pumilus* SH‐B9 and *Bacillus australimaris* MCCC 1A05787 respectively. Strain 3A and strain 4F were 98.09% and 97.27% matching to *Chryseomicrobium amylolyticum* JC16 and *Chryseomicrobium imtechense* HTHB4. Strain 6C and strain 6E were 99.29% identical to the *Arthrobacter koreensis* CA15‐8 and *Arthrobacter luteolus* LNR3 respectively. Strain 2C was 97.99% identical to the *Glutamicibacter mysorens* LMG16219. Strain 2D was 97.78% identical to the *Planococcus plakortidis* DSM 23997. The nucleotide sequences of the calcifying strains have been submitted to the NCBI database, and the accession number obtained is mentioned in Table S2. The class of *Bacillus, Arthrobacter* and *Planococcus* has been known for calcite production (Jonkers, 2011; Park *et al*., 2013; Mykytczuk *et al*., 2016). *Corynebacterium strain urealyticum* has been reported as calcifying strain (Cacchio and Del Gallo, 2019). However, the species of *Chryseomicrobium and Glutamicibacter* are first time identified as calcifying bacteria in the research. Figure 4 represents cladograms of isolated phylogeny. Inferred unrooted phylogenetic tree of calcifying microbes based on *16S rRNA* gene sequences isolated from soil, illustrating monophyletic, paraphyletic and polyphyletic relation of isolated strains. Tree indicated diversity in *16S rRNA*. There are two super clads comprising of three sub‐clads each. First super clad consists of *Arthrobacter*, *Glutamicibacter* and *Corynebacterium*. Other clad included *Bacillus, Planococcus* and *Chryseomicrobium*. The current study presented the diversity in genus of calcifying strains isolated from the local soils.

### SEM analysis of precipitated calcite

**Fig. 5:**
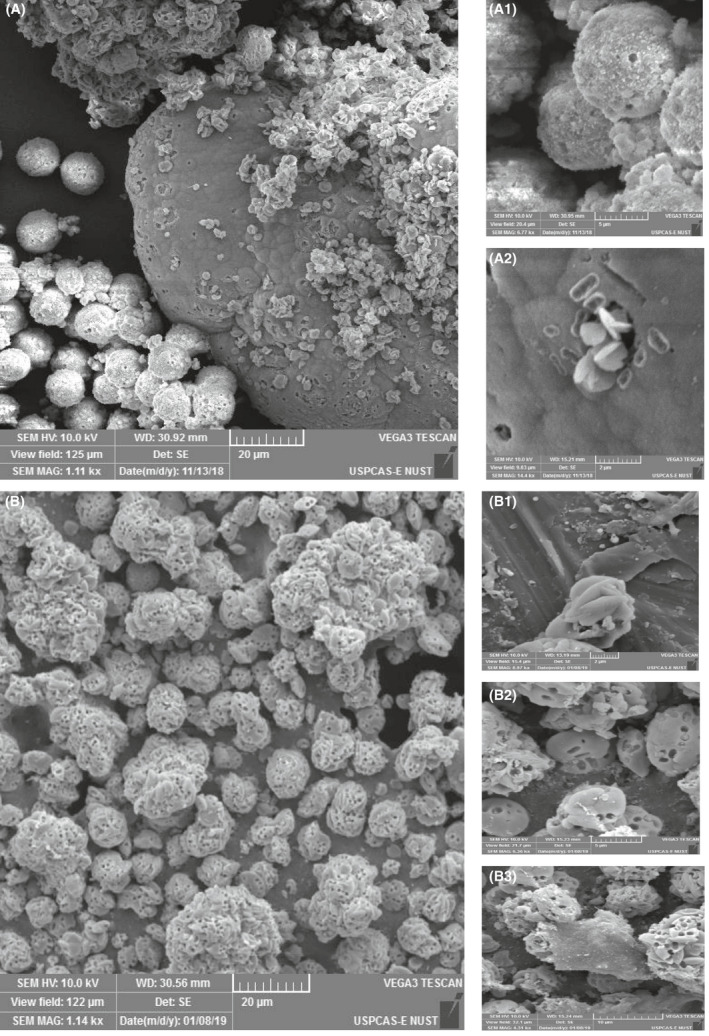
Effect of calcium source on calcite morphology; (A) calcite precipitates of calcium lactate source are mostly spherical vaterite (A.1) and rhombohedral calcite crystals (A.2); (B) calcite precipitates of calcium acetate source are mainly spherical oval vaterite (B.2) and few are rhombohedral (B.1) and amorphous CaCO_3_ (B.3).

**Fig. 6:**
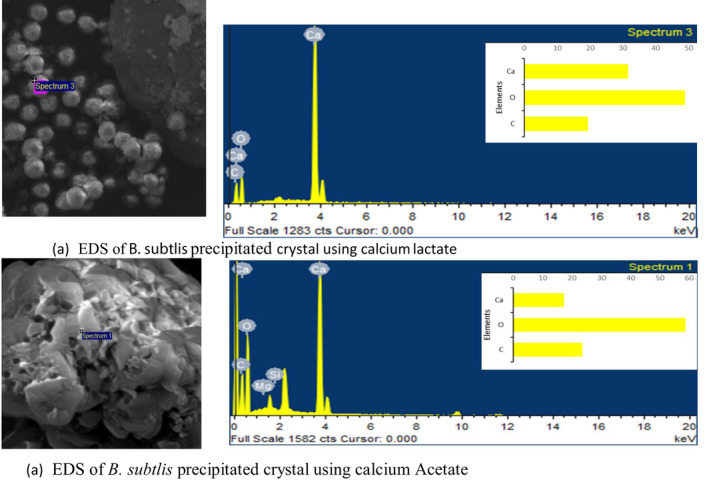
EDS of precipitated calcite.

Calcite precipitated from the bacterial strains was dried and examined under SEM complimented with EDS. Figure 5 represents the crystal morphology of CaCO_3_ by different calcium sources. The effect of calcium source on the morphology of CaCO_3_ is quite visible. It is also endorsed by the literature that bacterial type and calcium source influence the crystal morphology (Xu *et al*., 2015). Calcite, vaterite and aragonite are three different crystalline polymorphs of CaCO_3_ existing naturally (Seifan *et al*., 2017). In the current study, calcite and vaterite precipitation is observed. *B. subtilis* was used to rule out source effect on crystal precipitation. Precipitates of crystals using calcium lactate as source are spherical and rhombohedral crystals as shown in Fig. 5A. Spherical crystals are vaterite, while rhombohedral crystals are calcite (Al Omari *et al*., 2016). Vaterite is most unstable form of CaCO_3_ which eventually converts into calcite with time (Konopacka‐Łyskawa, 2019). Calcium acetates as a source precipitated crystals are mainly oval lens (spherical vaterite) like shape, but a few are rhombohedral (calcite) and some amorphous calcite is present (Fig. 5B) (Andreassen *et al*., 2012). The size of calcium acetates precipitates was larger than calcium lactate precipitates. Additionally, calcium acetate has more bacterial imprints than calcium lactate. This implies that calcium lactate precipices are dense and harder than calcium acetates. The presence of more vaterite than calcite is due to the high pH of the solution (Konopacka‐Łyskawa, 2019). For further confirmation of CaCO_3_, EDS of precipitated crystals was performed as depicted in Fig. 6. Results of EDS also endorsed the precipitation of CaCO_3_ as calcium content in both spectrums.

**Fig. 7:**
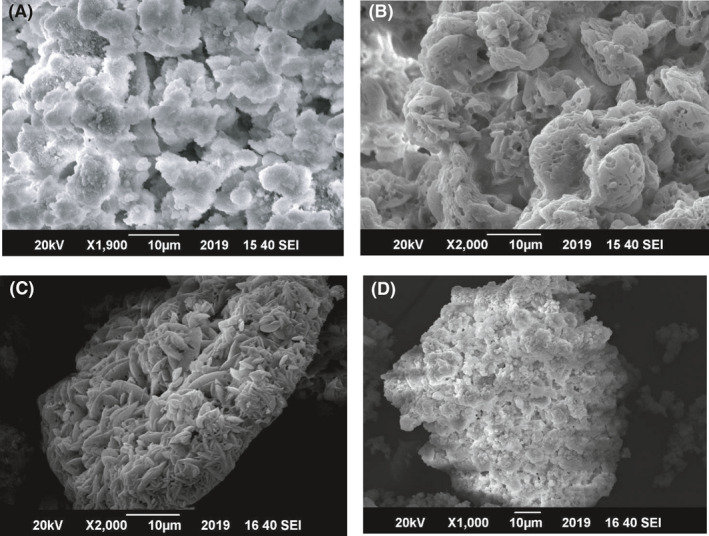
SEM analysis of precipitated calcite by (A) *B. safensis,* (B) *C*. *imtechense,* (C) *A. luteolus* and (D) *G. mysorens* using calcium lactate source.

As bacterial strain types effect the morphology of crystals (Wei *et al*., 2015), SEM of four different calcifying strains was performed using calcium lactate as calcium source and micrographs are displayed in Fig. 7. *B. safensis* (1G) and *G. mysorens* (2C) gave similar types of crystals which are irregularly stacked and very dense without any bacterial imprint and identified morphology is amorphous CaCO_3_ and similar structure was reported in literature using *Bacillus sp*. (Kim *et al*., 2016). *C*. *imtechense* (4F) gave higher bacterial imprint having a rhombohedral structure, and *A. luteolus* (6E) also depicted a dense pattern of crystals. These strains’ biogenic calcite is reported first time in the current study.

### Characterization of CaCO_3_ crystal structure of calcite by XRD and TG

**Fig. 8:**
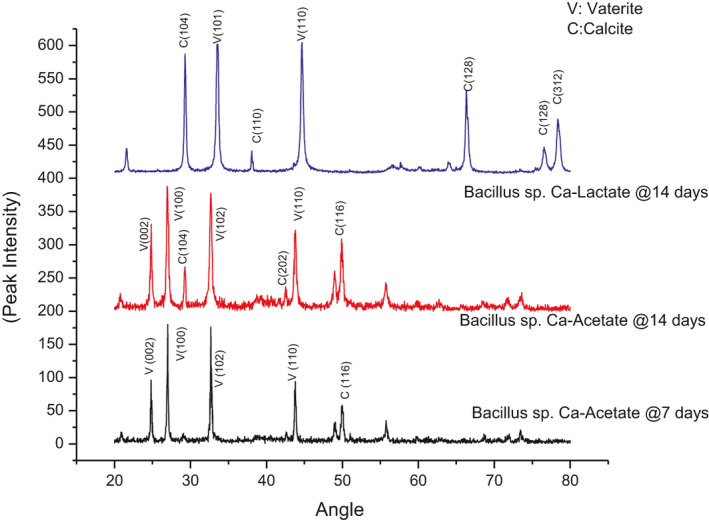
XRD pattern of precipitated calcite using different calcium sources.

X‐ray diffraction is a qualitative technique used to identify the crystal structure of a chemical compound. Distinctive peaks and corresponding orientation value (2*θ*) were recorded in the range of 5°–70° at the wavelength of 1.5Å. X‐ray diffractogram of precipitate is illustrated in Fig. 8. Most intense peaks obtained from the analysis resemble closely with the peaks of calcite and vaterite suggested by Al‐Jaroudi *et al*. (2007). Vaterite and calcite precipitation is bacterial strain‐ and calcium source‐dependent (Xu *et al*., 2015). Likewise, solution saturation and pH values play a major part in crystallization of these polymorphs (Andreassen, *et al*., 2012). Calcite is the most stable form of calcium polymorphs. However, vaterite converts into calcite with time (Wang and Becker, 2009). Conversion of some fraction of vaterite into calcite was evident after 14 days of incubation as compared to 7 days of incubation. In case of calcium lactate, the most dominant peak was obtained at 2θ value of 29.57° which is very close to 29.3°, 2θ value of pure calcite reported by Belcher et al. (Belcher et al., 1996).

**Fig. 9:**
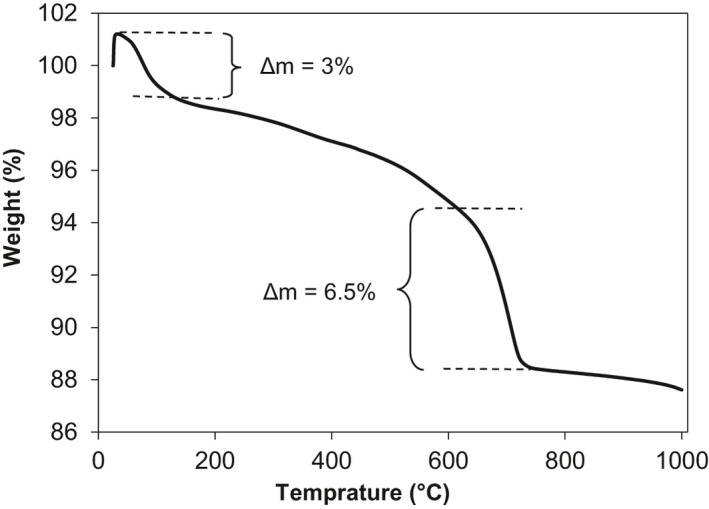
Thermal degradation curve of precipitated CaCO_3_ powder showing weight loss in the range of 600 to 800ºC.

CaCO_3_ decomposes thermally into CaO and CO_2_ at the temperature range of 600–850°C (Li *et al*., 2017). Carbon dioxide which is in gaseous form releases during this process resulting in significant weight loss. Results obtained from thermogravimetric analysis of the healing compound showed 38.97% weight loss in the specified temperature range of 600 to 850ºC. A sharp fall in weight starting at the temperature of 600°C is observed in Fig. 9, indicating the decomposition of CaCO_3_ into CaO and CO_2_ as reported by Kasselouri et al. (Kasselouri *et al*., 1995). This affirms the results obtained from XRF chemical analysis that healing compound mostly comprises of CaCO_3_ crystals.

### Mechanical evaluation of cementitious system

**Fig. 10:**
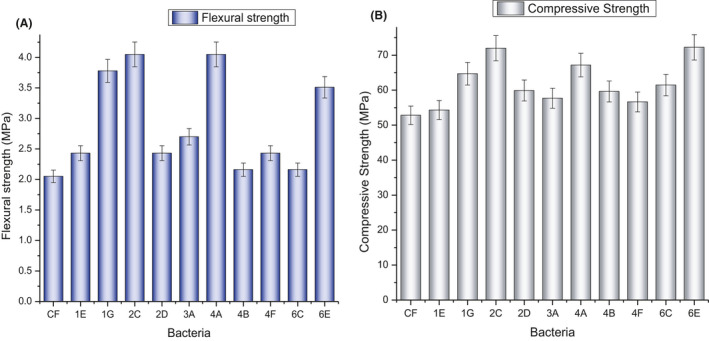
Mechanical properties of calcifying bacterial formulations. A. Flexural strength and (B) compressive strength.

Results of the flexural and compressive strength of specimens at 28^th^ day are presented in Fig. 10. An increase in the flexural and compressive strength was observed for all formulations. In the case of flexural strength, formulations containing *B*. *pumilus* (4A) and *G. mysorens* (2C) depicted 96% increment in flexural strength followed by *B. safensis* (1G) with 84% increase. *A. luteolus* (6E) depicted 72% increment in tensile strength, while 37% increase was observed in *C. amylolyticum* (3A) formulation. 18% increase was attained in formulation containing *C. efficiens* (1E), *P. plakortidis* (2D) and *C. imtechense* (4F). Similarly, 5% increment in flexural resistant was recorded in case of *B. australimaris* (4B) and *A. koreensis* (6C). A significant increase in flexural strength is attributed to bio‐calcite deposition as reported by Arthi & Dhaarani (2016). It is pertinent to mention that *Bacillus species* were only investigated in past literature and 49.4% increment in flexural strength was observed (Ghatiya and Pendharkar, 2017). However, the recent work adds the novel findings pertaining to *Arthrobacter, Planococcus, Chryseomicrobium* and *Corynebacterium* species.

A maximum of 36 % increase was observed in compressive strength in the formulation of *G. mysorens* (2C) and *A. luteolus* (6E). Almost 27% and 22% increase in compressive strength was observed in the case of *B. pumilus* (4A) and *B. safensis* (1G) respectively. *A. koreensis* (6C), *B. australimaris* (4B) and *P. plakortidis* (2D) showed increase by 16%, 13% and 12% in compressive strength. Similarly, 9%, 7% and 2% increment was recorded in the case of C. amylolyticum (3A), *C. imtechense* (4F) and *C. efficiens* (1E) respectively. The strength improvement mechanism is associated with the pore refinement via calcite precipitation (Khushnood, et al., 2018). *In vitro* results of calcite precipitation (Fig. 3) are comparable to the *in vivo* precipitation in mortar (Fig. 10) and can be endorsed via literature. The *Bacillus species* have been reported with maximum of 25.93% increase in compressive strength of cementitious mortar (Ghatiya and Pendharkar, 2017). *Bacillus species* are extensively researched in self‐healing concrete; however, isolated strains in the recent studies have not been reported yet (Khushnood et al., 2018; Khushnood *et al*., 2019; Shaheen *et al*., 2019). *Arthrobacter* specie is also reported as 8.9% strength increase in cementitious mortar (Park *et al*., 2010). *Planococcus* and *corynebacterium* strains have been reported as calcifying bacteria but never investigated in conjunction with cementitious mortar (Braissant *et al*., 2003; Dhami *et al*., 2017; Cacchio and Del Gallo, 2019). *Chryseomicrobium and Glutamicibacter* genera belonged to the Familia of *Planococcus* & *Arthrobacter;* however, available literature lacks in their calcification capability and is hereby publicized for the first time as calcifying strains.

Output data's significance was evaluated using t‐test. Data were significant having *P*‐values of 0.002046 and 0.000622 in case of flexural and compressive strength respectively.

## Conclusion

In this study, a rarely explored active pathway of CaCO_3_ precipitation (oxidation of organic matter) was adopted for screening the alkaliphilic calcifying microbes as an alternative approach to eliminate any toxic ammonia and sulfide formations. CaCO_3_ quantification protocol was established successfully to ascertain the most proficient and high endurance calcifying microbes at varying pH. Three of identified gram‐positive strains were spore former, while others were non‐spore formers. Most of these microbes did not show any urease hydrolysis. Prevailing species of identified strain were belonged to *Bacillus group*. *Bacillus safensis* gave an increase of 24.32% in calcite precipitation at pH 10, while *Arthrobacter luteus* depicted 58.2% increase than reference *Bacillus subtilis* at pH 7. Calcifying strains increased the system pH during precipitation process. Precipitated powder was characterized as polymorph of CaCO_3_ namely vaterite and calcite through XRD, SEM, EDS and TG. These strains were further investigated in the cementitious mortar for mechanical evaluation. All strains depicted higher mechanical strength than control formulation. A maximum of 96% increase in flexural strength was observed, while 36% was witnessed in case of compressive strength. *Bacillus* and *Arthrobacter* species gave higher mechanical strength than rest of the species. Experimental output data were significant having *P*‐values < 0.05 at the confidence interval of 95%. It is pertinent to mention that *Bacillus*, *Arthrobacter* and *Planococcus species* have been reported as CaCO_3_ producers but *Chryseomicrobium* and *Glutamicibacter* are reported first time in the current research as CaCO_3_ precipitators. These isolated strains have potential to enhance the resilience of cementitious matrix owing to their alkaliphilic nature and calcite precipitation aptitude through environmentally friendly pathway than urea‐based precipitation. Continuous pH change monitoring during the precipitation process is recommended for future work.

## Experimental procedures

### Soil sample collection

Soil samples were collected from six different locations of Pakistan including locality of iron industry S_1_ (Faisalabad, 31.4504° N, 73.1350° E), slum waste S_2_ (Faisalabad, 31.4504° N, 73.1350° E), locality of fabric industry S_3_ (Faisalabad, 31.4504° N, 73.1350° E), clinker soil S_4_ (Hatter, 30.297° N, 73.058° E), locality of cement quarry S_5_ (Hatter, 30.297° N, 73.058° E) and marble waste S_6_ (Hatter, 30.297° N, 73.058° E). Soil was collected at a depth of 20 cm after removing topsoil layers using sterile chisels, spatula and forceps and stored in sterile 50 ml Falcon™ tubes for transportation to the research laboratory for further microbiological, physical and chemical analysis. Moisture content was determined using established Eq. 5 reported in the literature (Rahman, *et al*., 2012). For *in vitro* pH measurement, 10 g soil was mixed with 100 ml water in flask and shaken for 45 min via roller shaker and filtered. Filtered water pH was measured using laboratory pH meter (Gregorich and Carter, 2007). For chemical analysis of soil, X‐ray fluorescence technique was used and palletization of soil samples was performed using hydro‐press at 40 psi pressure.

(5) $$\text{MC(\%) =}\;\frac{W2‐W3}{W3‐W1}\times 100$$

where

W1 = Weight of tin (g)

*W2 = Weight of moist soil + tin (g)*

*W3 = Weight of dry soil + tin (g)*

### Isolation of alkaliphilic bacteria

For isolation of alkaliphilic bacteria, LB agar media with pH 10 was used. Soil samples were serially diluted and spread on pH 10 agar plates. After overnight incubation at 37ºC, alkaliphilic bacterial colonies were collected and purified.

### Screening for calcifying bacteria

Purified strains were screened for calcite production using calcium precipitated media (CPM). As the focus of this study was the isolation of calcifying bacteria through oxidation of organic solids, modified B4 media was used as CPM that was comprised of (80 calcium lactate, 4 yeast extract, 15 agar) g per litre at pH 10 (Cacchio *et al*., 2003). In this study, calcium lactate was used in the replacement of calcium acetate and calcium chloride in B4 media. Optical density (OD) of overnight alkaliphilic bacterial strains was adjusted at 1.0 at 600 nm wavelength. Each bacterial culture (40 µl) was inoculated at the surface of modified B4 media plate. The plates were left undisturbed for 1–2 h, sealed and incubated at 37ºC for 15 days. The white powdery halo zone around the inoculated area was scraped off using a spatula and tested for calcite precipitation. *Bacillus subtilis* was used as a control for the identification of calcite precipitation.

A stationary broth test was also done in parallel to screen calcifying bacteria. For this, 15 ml falcon tubes were filled with 11 ml of 24 h bacterial culture (OD adjusted at 1.0) and 4 ml CPM broth media (Bhaduri *et al*., 2016). Falcons were incubated for 28 days at 37ºC and filtered with Whatman filter paper No. 1 and dried. Calcifying crystals were observed under an optical microscope (Marvasi *et al*., 2012). The results of both tests were compared and calcifying microbes were identified.

### Chemical verification of precipitated calcite

Precipitated calcite was initially verified by chemical methods. Acetic acid was added in dried calcite powder under fume hood and observed for effervescence as acetic acid breaks down into carbonic acid which further releases CO_2_. Further, calcite powder was boiled and added in hydrochloric acid (HCl), as HCl dissolves calcite powder.

### Quantification of calcite precipitation under different calcium sources

To quantify the relative calcite production potential of 24 of the selected calcifying strains under different calcium sources, i.e. calcium acetate and calcium lactate, a modified protocol given by Wei *et al.,* (2015) was adopted (Wei, *et al*., 2015). For that purpose, 48 ml CPM media (50 g calcium lactate/50 g calcium Acetate, 4 g yeast extract) g/litre at pH 10 and 2 ml calcifying bacterial culture were added in 100 ml Erlenmeyer flask and incubated at 37ºC and 200 rpm for 7 and 14 days. After incubation time, the media was poured in sterile falcon and centrifuged at 4000 rpm for 15 min. Pellet was resuspended in 50 ml TE buffer (pH 8) containing lysozyme (1 mg ml^‐1^) and kept for 1 h at room temperature. Lysozyme was added to digest bacterial cell debris. Centrifugation was carried out at 4000 rpm for 15 min and pellet was collected. Precipitated pellet was dried at 50ºC for 2 days and dried calcite powder was weighed. The experiment was done in triplicate and average was taken. *B. subtilis* was used as a reference control.

### Effect of varying pH on calcite precipitation

To check the effect of pH on calcite production, the selected 10 calcifying bacterial strains were inoculated in CPM media (calcium lactate as carbon source) at pH 7 and pH 10 for 14 days. The whole procedure was same as quantification of calcite. The experiment was done in triplicate and average was taken. *B. subtilis* was used as a control. pH change was examined at the end of the incubation period of 14 days.

### Characterization of calcifying bacteria

Gram staining of 10 selected calcifying bacterial strains was done to find out whether they are gram‐positive or gram‐negative bacteria. For understanding the biochemical activities, oxidase and catalase tests were performed. The oxidase test is used to identify the production of cytochrome C oxidase that is a mandatory enzyme of the bacterial electron transport chain. Catalase test is used to differentiate the bacteria that produce catalase enzyme from the non‐catalase producing bacteria. Catalase acts as a catalyst in the breakdown of hydrogen peroxide.

Urease test was performed to check the ability of calcifying bacteria to hydrolyse urea to produce ammonia and carbon dioxide. For that purpose, urease agar (Oxoid, UK) was autoclaved and filter‐sterilized urea 20 g l^‐1^ was added afterwards. Sterile 5 ml media was poured in test tubes and tubes were kept in slant position during cooling until solidified. Slant was streaked with fresh calcifying bacterial cultures and incubated at 37ºC. The slants were observed for a colour change at first at 6 h and then at the interval of 24 h up to 6 days.

### Spore Staining of calcifying bacteria

Spore staining was carried out to check whether selected calcifying bacteria are spore formers or non‐spore formers. For spore staining, bacterial cultures were grown for 72 h to induce sporulation. The slides of bacterial strains were prepared and stained with malachite green dye over steam for 3–5 min without boiling. Slides were rinsed with tap water and counterstained with safranin for 30 s (Bergey *et al*., 1994). Slides were washed off with tap water, blotted, air dried, and observed via microscope under oil immersion. *B. subtilis* was used as a positive control, while *E. coli* was used as a negative control.

### 16S rRNA sequencing of calcifying bacteria

DNA of the selected 10 calcifying bacteria was extracted through GeneJET Genomic DNA Purification Kit (Thermo Scientific™, Waltham, MA, USA). The protocol was used as recommended by manufacturer. The concentration of DNA was quantified through NanoDrop. The *16S rRNA* gene was amplified via PCR using universal primers 27F (5'‐AGAGTTGATCCTGGCTCAG‐3') and 1522R (5'‐AAGGAGGTGATCCAAGCCGCA‐3'). PCR was carried out using 2 µl DNA solution, 12.5 µl DreamTaq Green PCR Master Mix (Thermo Scientific™) and 10 pmol of each gene‐specific primer in a final volume of 25 µl.

PCR amplification conditions were as follows: initial denaturation step at 95°C for 5 min; 30 cycles of denaturation at 95°C for 30 s, annealing at 55°C for 40 s, extension at 72°C for 1 min, followed by a final extension step at 72°C for 10 min. The PCR product was sent to Macrogen, Korea (10F, 254 Beotkkot‐ro Geumcheon‐gu, Seoul, Korea) for sequencing. Resulting sequences were sorted for sequence homology via BLAST. For accession number, sequence was submitted to NCBI GenBank. The phylogenetic tree was constructed using MEGA X software by neighbour‐joining method with a bootstrap value of 1000.

### SEM, XRD and TGA of precipitated calcite

Crystal’s morphology and mineralogy of precipitated calcite were confirmed and examined through SEM, EDS, TGA and XRD techniques. Samples were completely dried and gold‐coated via sputter coating under the vacuum chamber prior to SEM examination. Additionally, energy dispersive X‐ray spectrometry (EDS) mapping for precipitated calcite was done to confirm the presence of calcium. For XRD, diffractogram was drawn at 2θº from a range 10º to 70º degrees and the components of the sample were identified by comparing them with standards established by the International Center for Diffraction Data.

Thermal gravimetric analysis (TGA) is a heat deferential method to confirm the presence of any substance. Calcite powder was heated up to 1000ºC at the rate of 10ºC min^‐1^. Mass deferential was compared to standard calcite powder.

### Mechanical evaluation of the cementitious system

The effect of bacterial inoculation in the cementitious system was investigated through the determination of compressive and flexural stresses. Ordinary Portland cement Type‐I conforming to ASTM C150 (grade 53) was used along siliceous sand with fineness modulus and specific gravity of 2.02 and 2.65 in the preparation of cement mortar composite samples. The average particle size ‘D_50_’ of cement was 16.50 μm with a density of 3.16 g cm^‐3^. For this purpose, mortar specimens having dimensions (40 x 40 x 160 mm) were casted according to standard ASTM C348 (EN, 2005; ASTM, 2008). For the casting of a specimen, a mix proportion (1:2) was used having 0.34 of water to cement ratio. Super‐plasticizer was used as 1% by weight of cement.

A total of 11 formulations were casted. Control formulation without bacterial strain was designated with CF, while the other 10 formulations were casted with a 4% replacement of water by bacterial culture and designated via respective strain name. Three specimens were casted for each formulation and tested at 28th day for flexural and compressive strength investigations (C^348^–^14^, ^2017^; C^349^–^14^, ^2017^).

### Statistical analysis

Numerical output of experiments was verified statistically. Analysis of variance (ANOVA) and *t*‐test using null hypothesis were performed alongside of standard deviations. Probability value less than 0.05 was considered statistically significant with confidence interval of 95%.

## Funding Information

For a part of tests, the laboratory and faculty support provided by ASAB and NICE at NUST are appreciated.

## Conflict of interest

The authors declare that they have no competing interest.

## Supporting information


**Fig. S1**. Spore former (left), Non‐spore former (right).
**Fig. S2**. Agarose gel amplified *16S* Microbial carbonate precipitation in construction materials *rRNA* gene product having amplicon size 1500 bp.
**Fig. S3**. (A) Abiotic control and isolated Strains precipitation after centrifuging to get pellet (B) After drying at 50ºC for 2 days.
**Table S1**. Physical and chemical properties of soil samples.
**Table S2**. Properties of selected calcifying bacterial strains.
