# Supplementary material for: Isolation of alkaliphilic calcifying bacteria and their feasibility for enhanced CaCO3 precipitation in bio‐based cementitious composites
Source: Microb Biotechnol. 2021 Feb 25;14(3):1044–59. doi: 10.1111/1751-7915.13752 (PMC8085925; doi:10.1111/1751-7915.13752)
Supplement: Supplementary file 1 — Fig. S1. Spore former (left), Non‐spore former (right). Fig. S2. Agarose gel amplified 16S Microbial carbonate precipitation in construction materials rRNA gene product having amplicon size 1500 bp. Fig. S3. (A) Abiotic control and isolated Strains precipitation after centrifuging to get pellet (B) After drying at 50ºC for 2 days. Table S1. Physical and chemical properties of soil samples. Table S2. Properties of selected calcifying bacterial strains. [file MBT2-14-1044-s001.docx]

Table 1: Physical and chemical properties of soil samples

| **Soil Sample** | **Locality of Iron industry** | **Slum waste** | **Locality of Fabric Industry** | **Clinker soil** | **Locality of Cement quarry** | **Marble waste** |
| --- | --- | --- | --- | --- | --- | --- |
| **Nomenclature** | S1 | S2 | S3 | S4 | S5 | S6 |
| **Moisture Content** | 8.17% | 2.95% | 1.92% | 5.54% | 16.1% | 28.4% |
| **pH** | 8.96 | 7.64 | 8.15 | 7.98 | 8.51 | 8.46 |
| **SiO_2_** | 19.9 | 40.5 | 26.4 | 29.98 | 28.5 | 13.1 |
| **CaO** | 8.87 | 4.7897 | 6.1 | 11.6 | 1.71 | 61.2 |
| **Fe_2_O_3_** | 56.12 | 52.7 | 58.4 | 53.95 | 64.8 | 7.5 |
| **MnO** | 0.21 | 0.34 | 0.22 | -- | 0.39 | -- |
| **TiO_2_** | 0.81 | 1.54 | 1.2 | 1.7 | 1.45 | -- |
| **SrO** | 7.24 | -- | 3.49 | 1.6 | 0.88 | 14.2 |
| **CuO** | 1.7 | -- | 0.45 | -- | -- | -- |
| **ZnO** | 2.49 | -- | 0.6 | 0.45 | 0.47 | -- |


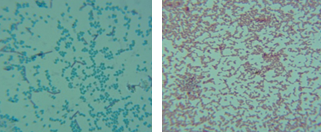


**Fig. 1:** Spore former (left), Non-spore former (right)


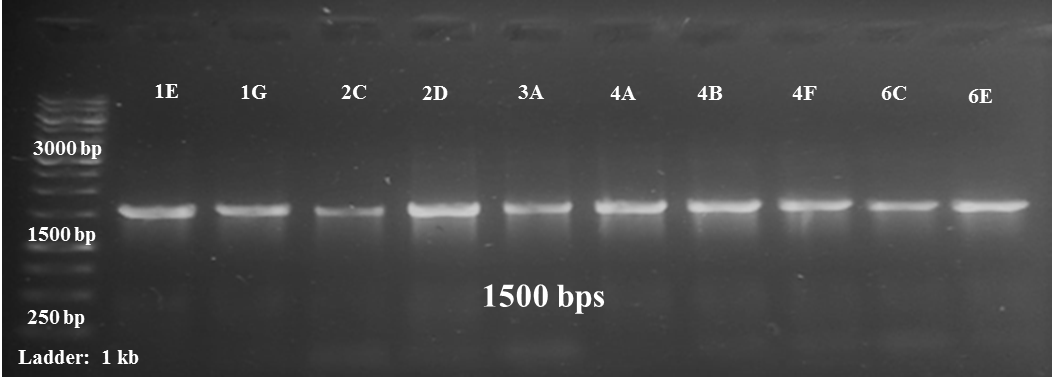


**Fig. 2.** Agarose gel amplified *16S rRNA* gene product having amplicon size 1500 bp


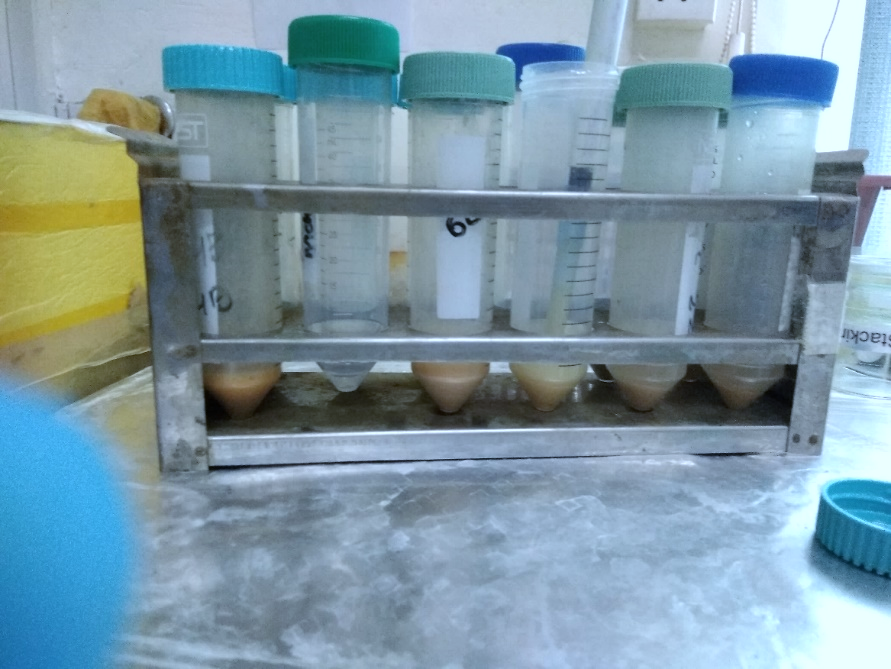

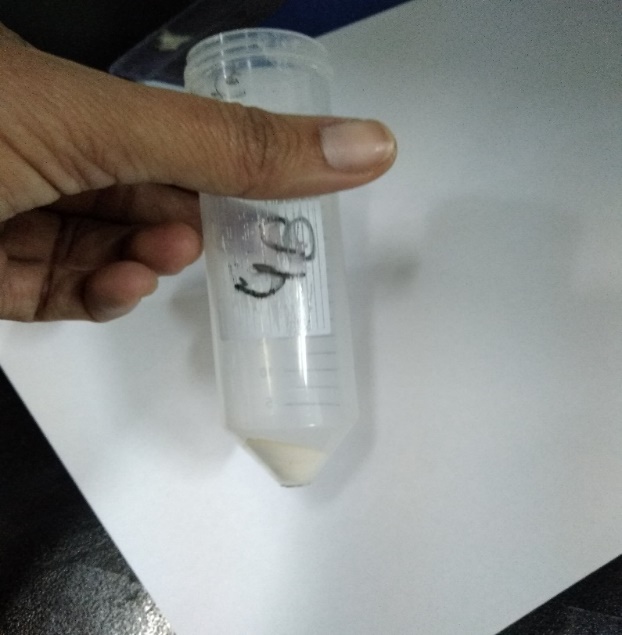


b

a

No Precipitation and cell debris in Abiotic control

Figure 3: (a) Abiotic control and isolated Strains precipitation after centrifuging to get pellet (b) After drying at 50ºC for 2 days

**Table 2:** Properties of selected calcifying bacterial strains

| **Property** | **1E** | **1G** | **2C** | **2D** | **3A** | **4A** | **4B** | **4F** | **6C** | **6E** |
| --- | --- | --- | --- | --- | --- | --- | --- | --- | --- | --- |
| **Colony color** | pale yellow | white | orange | Orange | Orange | white | white | orange | orange | orange |
| **Shape** | rod | rod | cocci | Cocci | cocci | rod | rod | Cocci | cocci | cocci |
| **Gram staining** | +ve | +ve | +ve | +ve | +ve | +ve | +ve | +ve | +ve | +ve |
| **Spore staining** | -ve | +ve | -ve | -ve | -ve | +ve | +ve | -ve | -ve | -ve |
| **Oxidase test** | -ve | +ve | -ve | -ve | -ve | +ve | +ve | -ve | -ve | +ve |
| **Catalase test** | +ve | +ve | +ve | +ve | -ve | +ve | +ve | -ve | +ve | +ve |
| **Urease test** | +ve | -ve | -ve | -ve | -ve | -ve | -ve | -ve | -ve | -ve |
| **Accession number** | MN865795 | MN865802 | MN865826 | MN955850 | MN865977 | MN865840 | MN865842 | MN867026 | MN865864 | MN865845 |
| **Query cover** | 100% | 100% | 99% | 98 | 99% | 100% | 100% | 100% | 100% | 100% |
| **Similarity index** | 98.18% | 98.92% | 97.99% | 97.78% | 98.09% | 99.44% | 99.63% | 97.27% | 99.29% | 99.29% |
| **Bacterial isolate** | *Corynebacterium efficiens* | *Bacillus safensis* | *Glutamicibacter mysorens* | *Planococcus plakortidis* | *Chryseomicrobium amylolyticum* | *Bacillus pumilus* | *Bacillus australimaris* | *Chryseomicrobium imtechense* | *Arthrobacter koreensis* | *Arthrobacter luteolus* |
